# A preview of selected articles

**DOI:** 10.1002/sctm.20-0565

**Published:** 2021-01-31

**Authors:** Stuart P. Atkinson

**Affiliations:** ^1^ Centro de Investigación Príncipe Felipe Valencia Spain

As the gold standard for the treatment of Parkinson's disease, Levodopa (or l‐DOPA) therapy can provide relief from motor symptoms for several years; however, this approach fails to completely reverse motor fluctuations and dyskinesias, and symptoms soon reappear. The grafting of dopamine neurons to replace the lost cells in the substantia nigra represents an alternative therapeutic strategy with the potential to addresses motor symptoms in the long term. This approach has been supported by the development of effective protocols for the differentiation of human pluripotent stem cells into midbrain dopamine (mDA) neurons.^1^, ^2^, ^3^ Importantly, the treatment of Parkinson's disease with differentiated mDA neurons, as with other pluripotent stem cell‐based approaches, suffers from safety problems related to the possible transplantation of surviving undifferentiated cells that may trigger unwanted growth/tumorigenesis. The targeting and activation of adult neurogenic niches represents a potentially safe and effective alternative to cell transplantation; however, we first require a deeper understanding of the processes controlling adult neurogenesis before we can exploit the induction of endogenous repair mechanisms as a treatment strategy. In our first Featured Article published this month in *STEM CELLS Translational Medicine*, Hiller et al report a protocol for the differentiation of human induced pluripotent stem cells (iPSCs) into mDA neurons for Parkinson's disease treatment that employs mitomycin‐C to reduce undesirable proliferation after transplantation.^4^ In a Related Article published recently in *STEM CELLS*, Weselek et al established the neurotransmitter norepinephrine as a negative regulator of adult neurogenesis in a study that highlights a possible means to stimulate cell regeneration in conditions such as Parkinson's disease.^5^

In patients with type 1 diabetes, the immune system attacks the insulin‐secreting β cells in the pancreas, which eventually induces β cell death and insulin deficiency. Therapies for the millions of type 1 diabetes patients worldwide include a modified diet, insulin injections to control glucose levels, immunotherapy, and pancreas/islet transplantations; however, insulin administration is not curative, and pancreas/islet transplantation has demonstrated only limited success to date. Notably, a huge body of preclinical research supports the treatment of type I diabetes with mesenchymal stem cell (MSC) therapy, with clinical trials now also evaluating the safety and efficacy of MSC administration in patients with new‐onset disease^6^ or in patients with chronic pancreatitis undergoing autologous islet transplantation.^7^ Given this burgeoning success, many have evaluated the potential of cell engineering to improve the therapeutic output of MSCs, while, in complementary research, others have sought to understand how type I diabetes negatively impacts stem cell therapies to then devise strategies to overcome this potentially damaging effect. In our second Featured Article published this month in *STEM CELLS Translational Medicine*, Song et al demonstrate that MSCs overexpressing anti‐inflammatory alpha‐1 antitrypsin (AAT) display improved efficacy with regards to the prevention of disease onset in a mouse model of type 1 diabetes.^8^ In a Related Article published recently in *STEM CELLS*, Chambers et al reported that myeloid angiogenic cells (MACs) display lower reparative and increased inflammatory potential under diabetic conditions thanks to the critical role of Interleukin 1 beta (IL1β), an essential mediator of the inflammatory response.^9^

## FEATURED ARTICLES

### Mitomycin‐C Treatment Improves the Safety of iPSC‐Derived mDA Neuron Transplantation

A recent study led by Jeffrey H. Kordower (Rush University Medical Center, Chicago, Illinois) reported on the differentiation of mDA neurons from human iPSCs that survived transplantation into the striatum of a rat model of Parkinson's disease and rescued disease phenotypes.^10^ While this approach employed a genetic drug selection process to ensure safety, the authors recently evaluated nongenetic approaches to constrain the activity of potentially proliferative cells while also considering the requirements for industrial scale‐up. As described in their recent *STEM CELLS Translational Medicine* article,^4^ Hiller et al screened mitotic inhibitors, DNA synthesis inhibitors, DNA cross‐linkers, and other selective agents with the potential to prevent aberrant cell growth during the large‐scale generation of mDA neurons from iPSCs. This screening effort revealed that sustained exposure to a low concentration of the DNA cross‐linker mitomycin‐C^11^ failed to influence the generation of mDA neurons from iPSCs, but did significantly reduce the number of proliferating cells after mDA neuron transplantation into an athymic rat model of Parkinson's disease. The use of mitomycin‐C also failed to impact grafting or survival of mDA neurons, and the study noted the recovery of lost function at a level comparable to that observed following the transplantation of mDA neurons derived using genetic drug selection. Overall, the authors provide evidence for mitomycin‐C treatment as an efficient nongenetic methodology to reduce undesirable proliferation following the transplantation of iPSC‐derived mDA neurons and improve the safety profile surrounding this potential effective Parkinson's disease therapy.

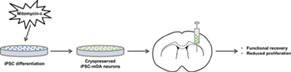

https://doi.org/10.1002/sctm.20-0014

### Alpha‐1 Antitrypsin Expressing MSCs as a Novel Diabetes Therapy

Given recent research demonstrating how the administration of AAT, an acute phase reactant and serine protease inhibitor that dampens inflammation, may represent an effective method to treat type 1 diabetes^12^ and improve islet survival and function after transplantation,^13^, ^14^ researchers from the laboratory of Hongjun Wang (Medical University of South Carolina, Charleston, South Carolina) sought to investigate the therapeutic potential of human bone marrow‐derived MSCs overexpressing AAT (AAT‐MSCs) in a type 1 diabetes mouse model. The team hoped that AAT expression might potentiate the immunomodulatory power of MSCs and permit the sustained suppression of the autoimmune response. Reporting in their recent *STEM CELLS Translational Medicine* study,^8^ Song et al discovered that AAT‐MSCs displayed improved innate properties in vitro, including enhanced self‐renewal, migrational, and multilineage differentiation abilities when compared with their parental MSCs and the overexpression of multiple genes related to stemness, migration, and survival. Moving in vivo, the authors demonstrated that a single intravenous infusion of AAT‐MSCs significantly limited any inflammatory cell infiltration into pancreatic islets and delayed disease onset in the type 1 diabetes mouse model when compared to mice treated with vehicle control or parental MSCs. In summary, this exciting study provides robust evidence that AAT overexpression may endow MSCs with the anti‐inflammatory/immunomodulatory power that could significantly improve the treatment of type 1 diabetes and other inflammatory diseases in human patients.

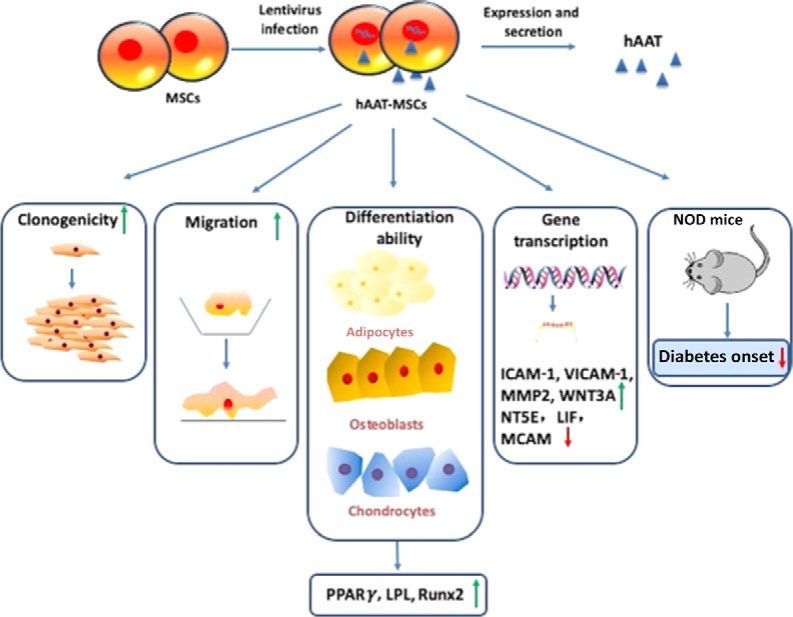

https://doi.org/10.1002/sctm.20-0122

## RELATED ARTICLES

### Norepinephrine as a Regulator of Adult Neurogenesis

A range of previous studies has highlighted the role of multiple neurotransmitter systems in the control of adult neural progenitor cell (NPC) proliferation within the subventricular zone of the lateral ventricles, thereby linking brain activity to neurogenesis.^15^ These findings prompted researchers led by Alexander Storch (University of Rostock, Germany) to explore the role of norepinephrine (NE, also known as noradrenaline^16^) as a regulator of NPCs within the subventricular zone of the adult mammalian brain in the hope of deepening our understanding of adult neurogenesis and informing on potential therapeutic options for neurodegenerative processes such as Parkinson's disease. In their recent *STEM CELLS* study,^5^ Weselek et al demonstrated that endogenous norepinephrine levels effectively inhibited NPC proliferation and neurogenesis within the adult mouse subventricular zone. While the intraventricular infusion of norepinephrine prompted a decrease in both NPC proliferation and neurogenesis (most likely mediated through the direct stimulation of β‐adrenoceptor signaling), the pharmacological inhibition of norepinephrine increased both NPC proliferation and the rate of early events associated with neurogenesis. In further confirmation, the neurotoxic ablation of norepinephrine neurons also increased NPC proliferation within the subventricular zone. Overall, the authors of this fascinating study provide evidence for norepinephrine as a physiological negative regulator of NPCs within the mouse subventricular zone and suggest a novel means to treat neurodegenerative conditions such as Parkinson's disease.

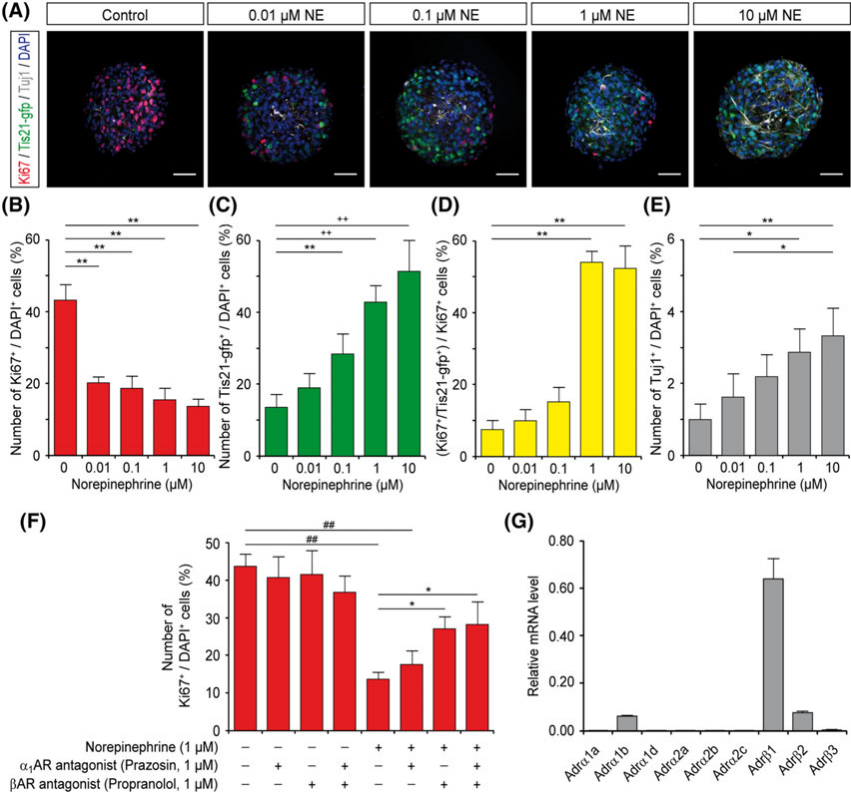

https://doi.org/10.1002/stem.3232

### Diabetes Impairs the Proangiogenic Properties of MACs

Previous research from the laboratory of Reinhold J. Medina (Queen's University Belfast, UK) described the isolation of macrophage‐like cells known as MACs from the mononuclear cell fraction of human peripheral blood and their full characterization.^17^, ^18^ Additionally, they demonstrated how MAC administration supported microvascular repair in a mouse model of retinal ischemia.^19^ As described in their more recent *STEM CELLS* article,^9^ Chambers et al next considered the potential impact of diabetes on the vasoreparative function of MACs and the mechanisms involved in any dysfunction, given that patients with type 1 diabetes generally suffer from severe vascular degenerative diseases. The authors evaluated MACs derived from type 1 diabetes patients or MACs exposed to a diabetic environment alongside normal controls; interestingly, they linked a significant reduction in the proangiogenic capacity of MACs exposed to diabetic conditions to the increased expression of the inflammatory mediator IL1β. However, the inhibition of IL1β via exposure to a neutralizing antibody significantly abrogated any diabetes‐induced antiangiogenic effects. Finally, the authors discovered that type 1 diabetes patients with microvascular complications displayed significantly higher levels of IL1β in MACs compared with type 1 diabetes patients not suffering from microvascular complications or nondiabetic patients. Overall, the authors hope that their description of how the diabetes‐induced upregulation of IL1β expression in MACs prompts a decrease in proangiogenic and regenerative capacities may allow for the development of novel therapeutic strategies that enhance the vasoreparative function of MACs.

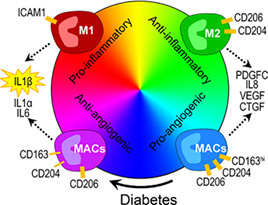

https://doi.org/10.1002/stem.2810
